# Supplementary material for: Information integration in large brain networks
Source: PLoS Comput Biol. 2019 Feb 7;15(2):e1006807. doi: 10.1371/journal.pcbi.1006807 (PMC6382174; doi:10.1371/journal.pcbi.1006807)
Supplement: S1 Text — (PDF) [file pcbi.1006807.s001.pdf]

Information Integration in Large Brain Networks

Daniel Toker[1*], Friedrich T. Sommer[1],

1 Helen Wills Neuroscience Institute, University of California, Berkeley, Berkeley, California, United States

* danieltoker@berkeley.edu

Supporting Information

Extrapolating Integrated Information to Infinite Observations

As we mentioned in the main body of the paper, estimates of information measures from finite time-series data are typically over-estimated. To get around this over-estimation problem, past work in information theory (1; 2; 3), inspired by earlier work in statistical mechanics (4), has produced a simple method for extrapolating information estimates to what they would be if infinite data were available. Though we did not utilize this extrapolation method in the main body of the paper, we present it here for two reasons: 1) we include the method in our Matlab toolbox, and 2) to demonstrate that it successfully mitigates the over-estimated results reported in Fig. 3 (i.e. the results from the structurally severed networks, for which the ground-truth value of integrated information should trivially be 0 bits).

Consider a jointly Gaussian variable, described by a linear regression model:

$$X_t = AX_{t-\tau} + E \tag{1}$$

where X_t is the present of the system, $X_{t-\tau}$ is the past of the system, A is the regression matrix, and E is the error or residuals in the linear regression. The geometric integrated information of a this Gaussian process can be calculated analytically without time-series data, by constructing a random regression matrix A. To do so, we simply create a random dense positive definite matrix. This can be done in Matlab using `sprandsym.m`. To ensure that the graph is fully connected, we set the density of the graph to 1 (otherwise the MIB will trivially separate disconnected nodes). Since the calculation of geometric integrated information assumes stationarity, we decay the coefficients of the random dense positive definite matrix so that its spectral radius is less than 1 (here, we pick a spectral radius of 0.8). This can be done with the `var_specrad.m` function of the MVGC Multivariate Granger Causality Matlab Toolbox (5).

For simplicity, we assume that the dynamics of the system are fully described by interactions between the variables and that "noise" in the system is uncorrelated. Thus, we set the elements along the diagonal of the covariance of the error matrix to 1, and set all off-diagonal elements to 0.

In this system, the covariance of the present state of the system can be computed by solving the discrete-time Lyapunov equation. In Matlab, this can be done with `dlyap.m` command. This gives us $\Sigma(X)$, and so we have everything we need to analytically calculate integrated information in this system. To do so, we iterate through all possible bipartitions of the regression matrix A, and for each candidate partition we set all cross-partition connections in the corresponding disconnected regression matrix A' to 0. We then use an augmented Lagrangian method to determine the non-zero values of A',

which then lets us analytically calculate the covariance matrix $\Sigma(E')$ of the residuals of the disconnected model. We further use $\Sigma(X)$ to analytically compute the differential entropy of each divided sub-system: if K is the submatrix of $\Sigma(X)$ that corresponds to one sub-community, then the entropy of that sub-community is $\frac{1}{2}\log[(2\pi\mathrm{e})^{\mathrm{n}}|\det(\mathrm{K})]]$, where n is the number of variables/nodes in the sub-community. We then use these entropy values to calculate normalized integrated information. We select the partition that minimizes normalized integrated information as the MIB. The non-normalized integrated information across that partition is the analytic ground-truth for this system.

We can now demonstrate the accuracy of our extrapolation method by generating time-series data for these systems. We do this by simply starting with random initial conditions, and, at each time step, we multiply the preceding values by the regression matrix and add Gaussian noise (which, by constructing $\Sigma(E)$ such that off-diagonal elements are 0, we ensure is uncorrelated).

With this multivariate time-series, we can compute integrated information following the same steps as those described in the main body of the paper. If the widely-used method for extrapolating entropy and mutual information to infinite observations (as described in the main body of the paper) works well for integrated information, then we should expect our extrapolated estimate of Φ^{G} to be close to the ground-truth in these systems.

We followed the above steps to generate analytic ground-truth values, time-series data, and extrapolated estimates of Φ^{G} in 14- and 16-node networks, for which the ground-truth MIB can be established through a brute-force search through all possible bipartitions of the randomly constructed regression matrices. We found that in these networks, the extrapolation method is highly successful, and provides values very close to the ground-truth (Figure S1A-C). Moreover, the majority of MIBs identified by extrapolating on the time-series data were identical to their corresponding ground-truth MIBs (determined by searching through all possible bipartitions of the ground-truth regression matrix) (S1D Fig).

Finally, we applied this extrapolation method to the simulated data generated from the structurally severed networks reported in Fig. 3. In principle, the most rigorous method of extrapolation (which was used in S1A-D Fig) would be to extrapolate to infinite observations for *every candidate bipartition*, and to pick as the MIB the bipartition that minimizes *extrapolated* normalized integrated information. This becomes very comutationally expensive for large networks, however, so for the following analyses we only extrapolated to infinite observations using the bipartition estimated from the finite data. (Note that our Matlab toolbox can handle either picking one partition and extrapolating on that partition, or extrapolating on all candidate partitions). We found that although estimates of Φ^{G} in the cut networks diverge from the expected ground-truth of 0 bits (S1E-F Fig), as is expected given the notorious over-estimation bias of information measures, extrapolation reduces this over-estimation bias and brings estimates closer to the ground-truth of 0 bits (S1G-H Fig).

References

1. Strong SP, Koberle R, van Steveninck RRdR, Bialek W. Entropy and information in neural spike trains. Physical review letters. 1998;80(1):197.

2. Brenner N, Strong SP, Koberle R, Bialek W, Van Steveninck RRDR. Synergy in a neural code. Neural computation. 2000;12(7):1531–1552.

3. Dayan P, Abbott LF. Theoretical neuroscience. vol. 806. Cambridge, MA: MIT Press; 2001.

4. Ma Sk. Calculation of entropy from data of motion. Journal of Statistical Physics. 1981;26(2):221–240.

5. Barnett L, Seth AK. The MVGC multivariate Granger causality toolbox: a new approach to Granger-causal inference. Journal of neuroscience methods. 2014;223:50–68.
