# Supplementary material for: Information integration in large brain networks
Source: PLoS Comput Biol. 2019 Feb 7;15(2):e1006807. doi: 10.1371/journal.pcbi.1006807 (PMC6382174; doi:10.1371/journal.pcbi.1006807)
Supplement: S1 Fig — (PDF) [file pcbi.1006807.s002.pdf]

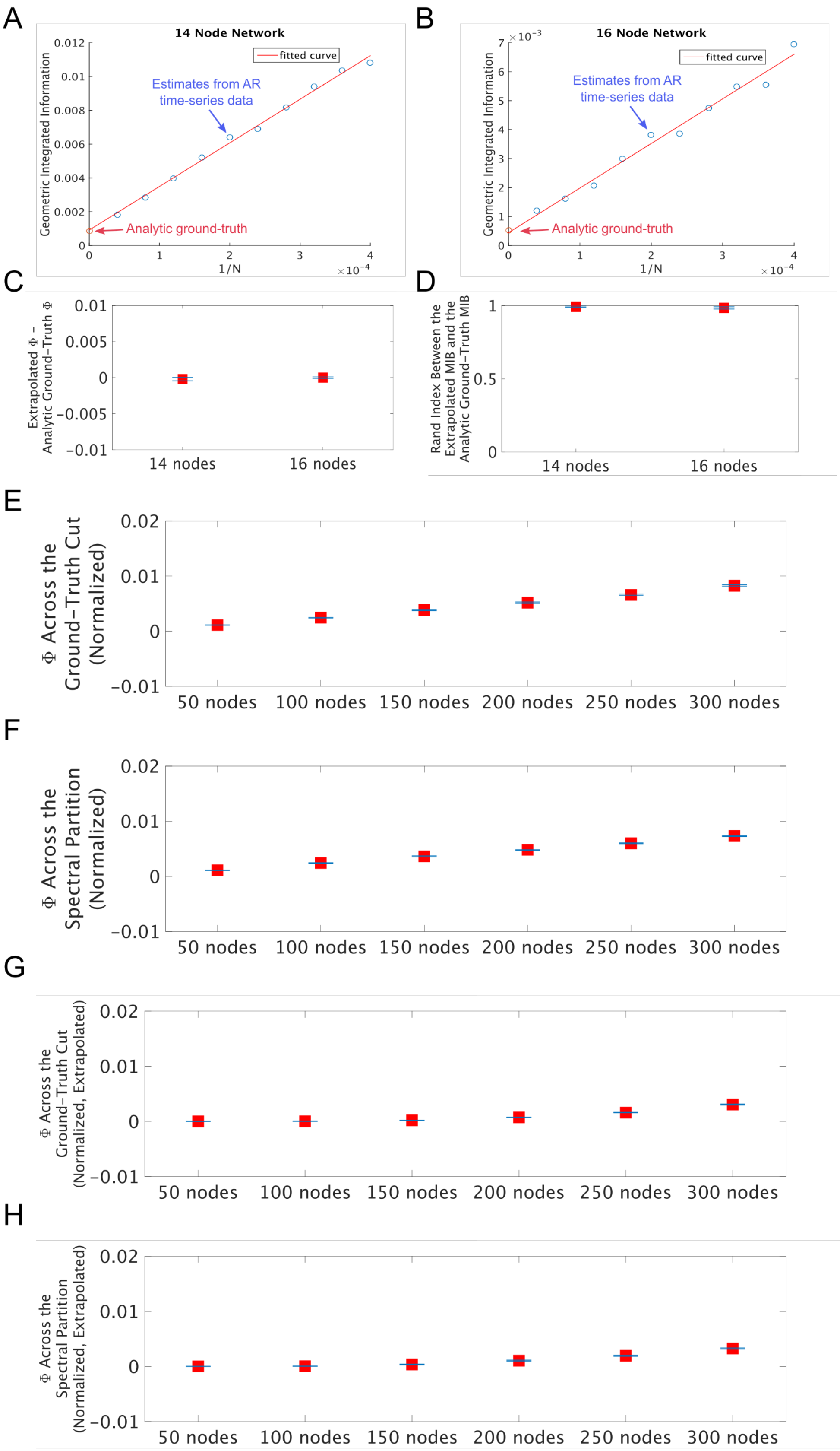

(Caption on next page.)

S1 Figure. Results of extrapolation analysis. In the absence of infinite data, integrated information can be accurately estimated from finite time-series data. Following well-established for estimating entropy and mutual information from finite time-series data, we calculate integrated information from time-series data as a function of $1/N$, where N is the number of observations. We then fit a line to this distribution of estimated values, and estimate integrated information as the value of the line at $1/N = 0$. **A** A sample of measurements of Φ^{G} as a function of 1/N in a 14-node autoregressive system. The orange circle on the y-axis is the analytic ground-truth value of Φ^{G} in this system, and the y-intercept of the line fitted to estimates of Φ^{G} from sub-sampled time-series data is the estimate of Φ^{G} extrapolated to infinite observations (i.e. where 1/N=0). Note that the value of the fitted line at $1/N = 0$ is remarkably close to the analytic ground-truth value. **B** A sample estimate of integrated information from a 16-node autoregressive system. **C** We generated autoregressive time-series data from 60 14-node networks and 60 16-node networks, and then compared our extrapolated estimates of integrated information to the analytic ground-truth for those systems. Our extrapolated estimates were highly accurate: the mean absolute error for the 14-node networks was 0.001 bits, and the mean absolute error for the 16-node networks was less than 0.0005 bits. Red squares are the mean across tested networks, and blue bars indicate standard error of the mean. **D** For our autoregressive time-series, we extrapolated integrated information to infinite observations across all possible bipartitions, and selected the bipartition that minimized *normalized* integrated information extrapolated to infinite observations as the MIB. We then compared these bipartitions to the analytic ground-truth MIBs. Our estimates based on time-series data found the exact (analytic) MIB in 57/60 of the 14-node systems (mean Rand Index = 0.99) and in 56/60 of the 16-node systems (mean Rand Index = 0.98). Thus, we can confidently assume that well-established extrapolation methods work for integrated information. **E** In the structurally severed networks in Fig. 3 of the main paper, there should trivially be 0 bits of integrated information. Yet, as we show here, estimates of Φ^{G} in these networks given finite time-series data often yield results greater than zero. In particular, larger networks seem to result in larger over-estimation of Φ^{G}. This same finding holds for non-extrapolated values across the bipartitions identified by our spectral clustering approach (**F**), which is not surprising, because our approach usually found partitions similar to the ground-truth cuts in these networks (Fig. 3). Using the extrapolation procedure described here, however, mitigates this over-estimation bias and brings estimates of integrated information close to the ground-truth of 0 bits (**G,H**).
