# Supplementary material for: Information integration in large brain networks
Source: PLoS Comput Biol. 2019 Feb 7;15(2):e1006807. doi: 10.1371/journal.pcbi.1006807 (PMC6382174; doi:10.1371/journal.pcbi.1006807)
Supplement: S2 Fig — (PDF) [file pcbi.1006807.s003.pdf]

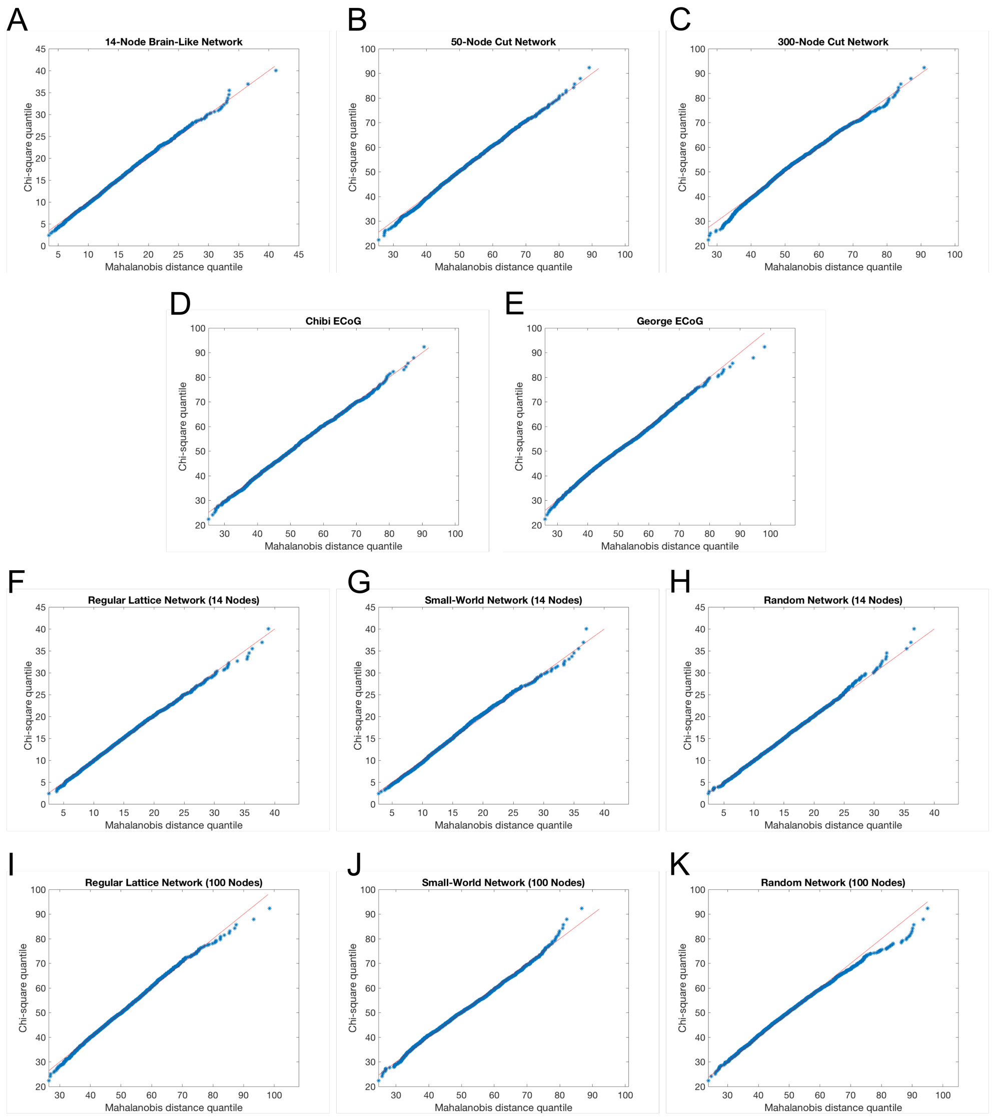

S2 Figure. Assessing data multivariate normality. All time-series data analyzed in this paper were approximately multivariate normal. This is important, because the estimator of geometric integrated information we used assumes multivariate normality. As a graphical test of multivariate normality, we used multivariate Q-Q plots: if data are multivariate normal, then there should be a linear relationship between the ordered Mahalanobis distances of the data from the mean vector and their corresponding chi-square quantiles. Here, we show sample multivariate Q-Q plots for our 14-node brain-like networks of coupled Rössler oscillators (**A**), our cut, 50-node brain-like networks of coupled Rössler oscillators (**B**), our cut, 300-node brain-like networks of coupled Rössler oscillators (**C**), the ECoG data from Chibi (**D**), the ECoG data from George (**E**), our 14-node regular lattice networks (rewiring probability p=0) of coupled Rössler oscillators (**F**), our 14-node small-world networks (rewiring probability p=0.5) of coupled Rössler oscillators (**G**), our 14-node random networks (rewiring probability p=1) of coupled Rössler oscillators (**H**), our 100-node regular lattice networks (rewiring probability p=0) of coupled Rössler oscillators (**I**), our 100-node small-world networks (rewiring probability p=0.5) of coupled Rössler oscillators (**J**), and our 100-node random networks (rewiring probability p=1) of coupled Rössler oscillators (**K**).
