# Supplementary material for: Information integration in large brain networks
Source: PLoS Comput Biol. 2019 Feb 7;15(2):e1006807. doi: 10.1371/journal.pcbi.1006807 (PMC6382174; doi:10.1371/journal.pcbi.1006807)
Supplement: S3 Fig — (PDF) [file pcbi.1006807.s004.pdf]

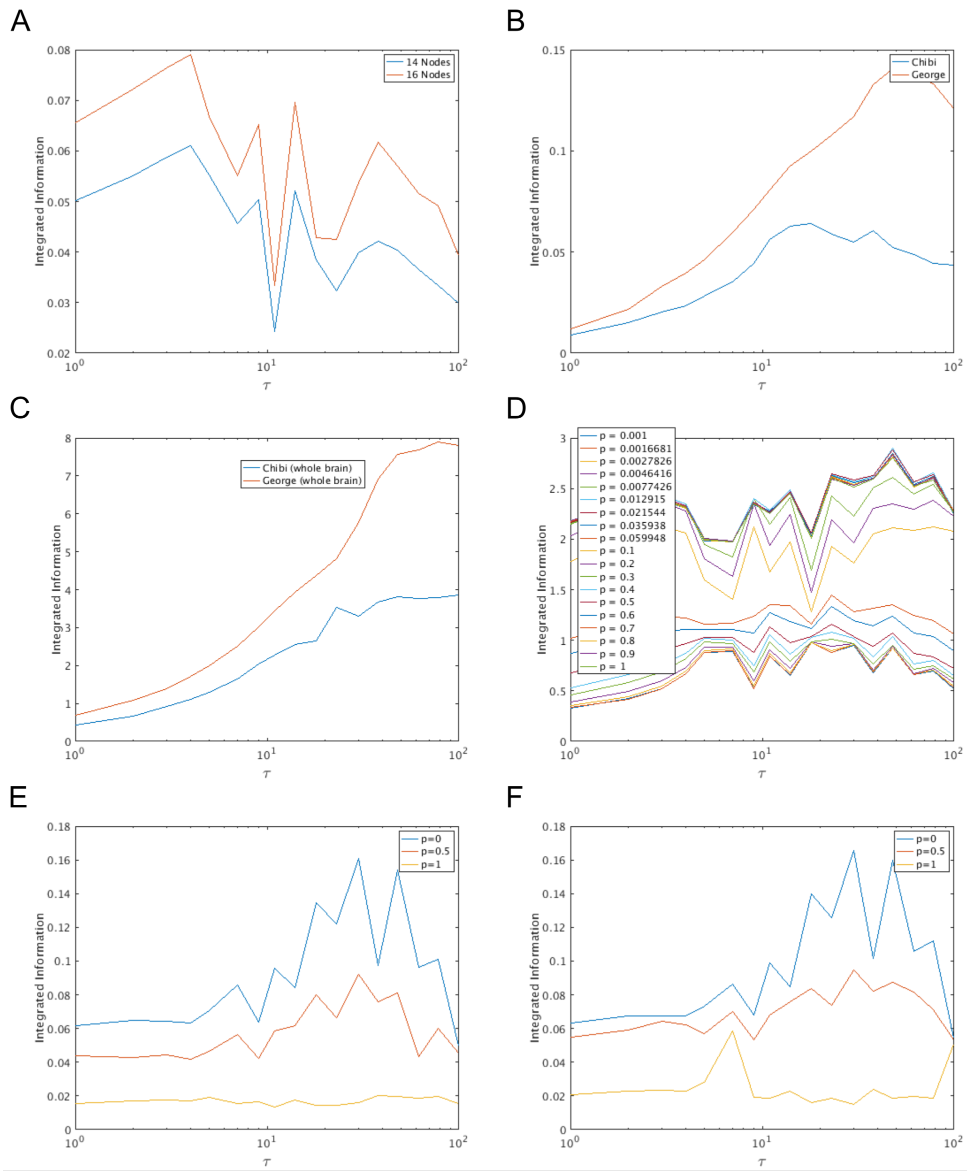

(Caption on next page.)

S3 Figure. Integrated information as a function of time-lag. Integrated information is measured over a time-lag τ. For all analyses in our paper, we picked a time-lag that, on average, maximized integrated information for the system at hand. To test a range of time-lag values, we measured integrated information across 17 different time-lags, logarithmically spaced between 1 and 100. For both our 14-node and 16-node brain-like networks of coupled Rössler oscillators, we were able to calculate integrated information across the MIB (estimated from finite data) for each candidate time-lag. Here we plotted the median values of integrated information across all simulated networks. We found that integrated information was typically maximized at a time-lag of 4 (**A**). For the 14-electrode monkey ECoG data, we picked a time-lag of 38, as the median integrated information as a function of time-lag across all 112 sets of electrodes per monkey seemed to peak or aymptote around 38 (**B**). For the whole monkey brains, we calculated integrated information using our spectral clustering-based approach, and found that our estimates peaked around a time-lag of 48, which is 96 ms. (**C**). We similarly calculated integrated information as a function of time-lag using our spectral clustering approach in our 100-node Watts-Strogatz networks (which ranged from regular lattice networks to small-world networks to random networks, depending on the value of rewiring probability p). Interestingly, we found quite different behaviors of integrated information as a function of time-lag for networks with a rewiring probability less than 0.1 than we did for networks with a rewiring probability greater than 0.1, but all networks peaked at a time-lag of 48, and so we chose a τ of 48 for the analyses in Fig. 5. (**D**). Finally, we calculated integrated information across the MIB in our 14-node Watts-Strogatz networks (**E**) and in our 16-node Watts-Strogatz networks (**F**), and found that the median integrated information peaked around a time-lag of 30. For the cut networks analyzed in Fig. 3, we used a time-lag of 1 beceause there is objectively no information integration in these networks, and thus the choice of time-lag should not matter.
