# Supplementary material for: Information integration in large brain networks
Source: PLoS Comput Biol. 2019 Feb 7;15(2):e1006807. doi: 10.1371/journal.pcbi.1006807 (PMC6382174; doi:10.1371/journal.pcbi.1006807)
Supplement: S4 Fig — (PDF) [file pcbi.1006807.s005.pdf]

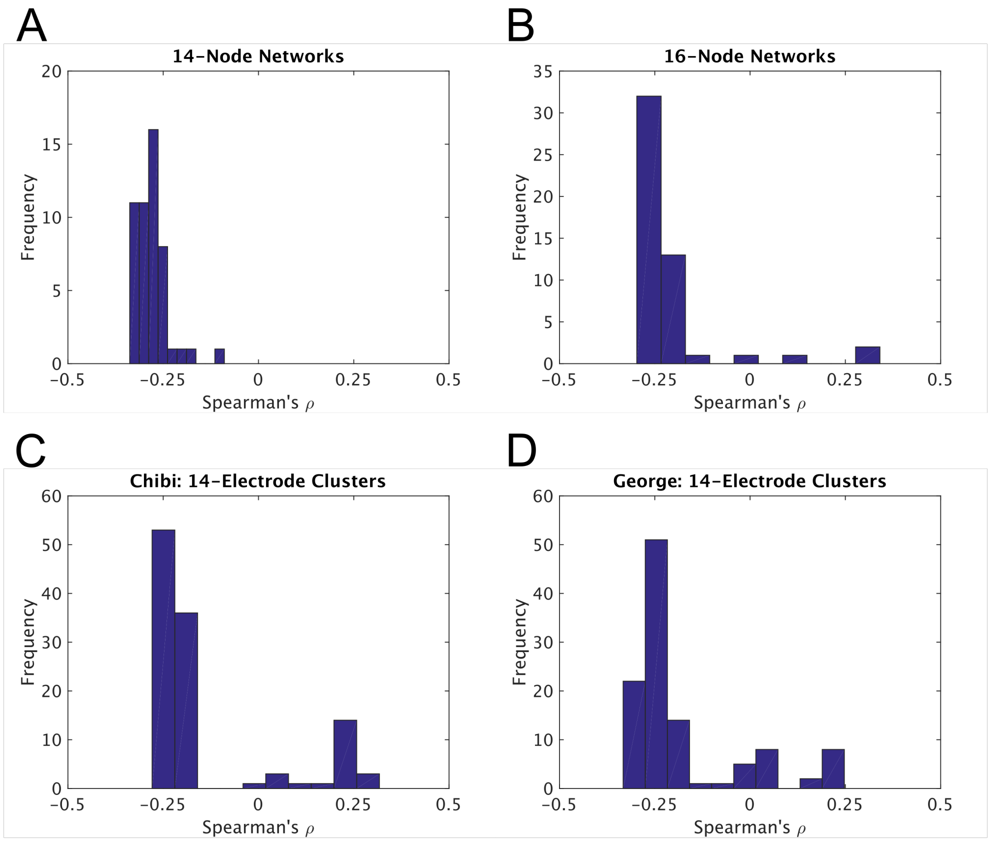

S4 Figure. Integrated information and partition similarity to the MIB. In general, but not always, the closer a partition is to a network's minimum information bipartition, the smaller integrated information (normalized) will be across that partition. Here, we calculated Φ^{G} (normalized) across every possible bipartition of every small dataset (up to 16 nodes) analyzed in the main body of this paper. We then computed the Rand Index between those bipartitions and the ground-truth minimum information bipartition of that dataset. If finding a partition close to the ground-truth MIB is important for accurate estimation of integrated information, then we should in general expect a negative correlation between Φ^{G} (normalized) across all possible bipartitions and the Rand Indices between those bipartitions and the ground-truth MIB (though in some networks there may be large local minima that are dissimilar to the MIB). In other words, the more dissimilar a partition is from the MIB, then the higher normalized integrated information across that partition should typically be (again, discounting the case of large local minima). Here, we find that this is generally the case in both real and simulated data. Because the distributions of Rand Indices were not normally distributed, we calculated the correlation between Rand Indices and normalized Φ^{G} values using a Spearman's rank correlation. We found negative Spearman's correlations, as expected, for most 14-node networks of coupled Rössler oscillators (**A**), 16-node networks of coupled Rössler oscillators (**B**), and 14-electrode clusters of ECoG data for both monkeys (**C, D**).
