# Supplementary material for: Information integration in large brain networks
Source: PLoS Comput Biol. 2019 Feb 7;15(2):e1006807. doi: 10.1371/journal.pcbi.1006807 (PMC6382174; doi:10.1371/journal.pcbi.1006807)
Supplement: S5 Fig — (PDF) [file pcbi.1006807.s006.pdf]

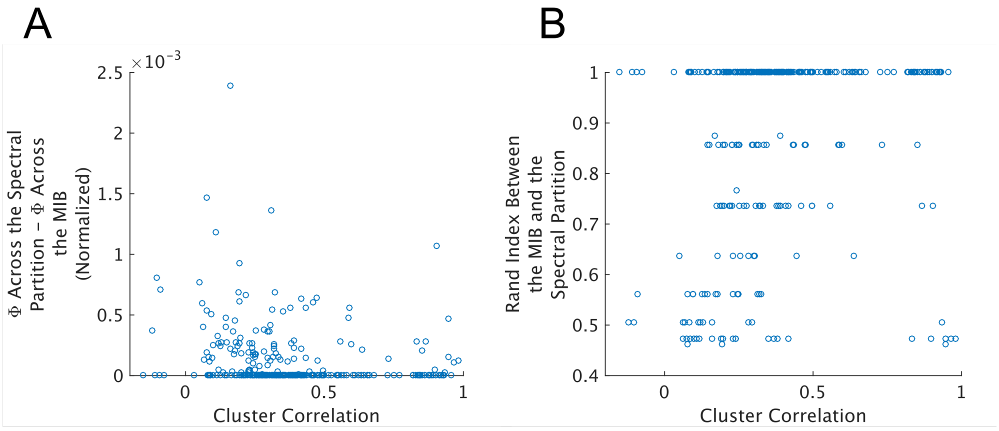

S5 Figure. Cluster correlation as a predictor of MIB estimation accuracy. Though our spectral clustering-based approach found the exact MIB in the majority of small datasets analyzed in this paper (for which the ground-truth can be computed through a brute-force search), it did not always find the exact solution. As we report in the main body of the paper, in the cases that spectral clustering did not find the exact MIB, it usually still found partitions close to the MIB, and yielded values of integrated information that were close to the ground-truth. But this presents an interesting problem: is there a way to predict how likely it is that spectral clustering found the right solution - or something near it - when the ground-truth is not known? Though we could not find a variable that was predictive of the success of spectral clustering in all cases, we did observe that the more correlated nodes were on either side of the partition, the more likely it was that spectral clustering had found the ground-truth MIB. Here, we capture that observation with a variable which we call "cluster correlation," which is the mean of all within-cluster correlations minus the mean of all cross-cluster correlations. In other words, if spectral clustering splits a network into clusters A and B, then the cluster correlation is the mean of the correlations between all nodes in A, minus the mean of correlations between nodes in A and nodes in B. In this analysis, we collapsed results across all datasets for which the ground-truth MIB could be observed (i.e., all 100 small simulated brain-like networks of coupled Rössler oscillators, and the 224 small sets of monkey ECoG electrodes). **A** We observed a general negative correlation (Spearman's ρ=-0.28, p< 10^{-6}) between the cluster correlation and the difference between integrated information (normalized) across the MIB and integrated information (normalized) across the spectral partition. In other words, the stronger the within-community correlations were relative to cross-community correlations, the closer Φ^{G} (normalized) across the spectral partition was to Φ^{G} (normalized) across the MIB. This is probably because community clustering is generally easier when there are strong within-community weights or correlations. In contrast, we did not observe a significant negative correlation (Spearman's ρ=-0.0265, p=0.64) between the modularity Q of correlation matrices split by spectral partitions and the difference between integrated information (normalized) across the MIB and integrated information (normalized) across spectral partitions, despite the intuitive similarity between modularity and our measure of cluster correlation. **B** Consistent with our results in **A**, there was a significant *positive* correlation (Spearman's ρ=0.29, p< 10^{-7}) between the cluster correlation and the Rand Index between the spectral partition and the MIB. Again, the modularity of the correlation matrices split by spectral partitions was not significantly correlated with the Rand indices (Spearman's ρ=0.06, p=0.28). These results suggest that spectral clustering provides the most accurate estimate of the MIB in networks whose time-series data produce a correlation structure in which within-module correlations are stronger than cross-module correlations. That said, we found that spectral clustering also usually finds the exact MIB in non-modular networks, such as regular lattice networks and random networks (S6 Fig).
