# Supplementary material for: Information integration in large brain networks
Source: PLoS Comput Biol. 2019 Feb 7;15(2):e1006807. doi: 10.1371/journal.pcbi.1006807 (PMC6382174; doi:10.1371/journal.pcbi.1006807)
Supplement: S6 Fig — (PDF) [file pcbi.1006807.s007.pdf]

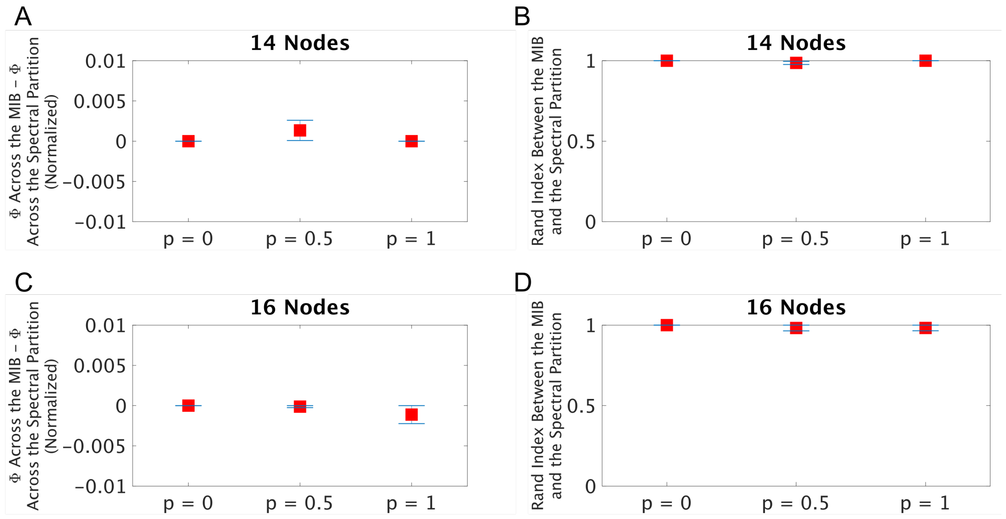

S6 Figure. Spectral clustering accuracy in Watts-Strogatz networks. Spectral clustering accurately identifies the MIB of a variety of network types. Here, as in Fig. 5 in the main body of the paper, we followed Watts and Strogaz's method for generating different network types by varying the rewiring probability p of lattice networks. A p of 0 yields a perfect lattice network, a p of 0.5 yields a small-world network, and a p of 1 yields a random network. Here, we generated 30 lattice ($p = 0$), 30 small-world ($p = 0.5$), and 30 random ($p = 1$) networks, of 14- and 16-nodes in size. With each of these networks, 25,000 time-points of oscillatory time-series data were generated using the stochastic coupled Rössler oscillator model, with the coupling coefficient set to 0.7 for all networks; these simulations yielded multivariate normal dynamics (Fig. S2F-H). We then compared estimates of integrated information across the MIB and across the spectral clustering-based partition for each of the resulting 180 data sets. We found that spectral clustering performed perfectly in all regular lattice ($p = 0$), with perfect matches to the MIB (**B,D**) and no difference from the ground-truth Φ (normalized) values (**A,C**) in all tested networks. For the small-world networks ($p = 0.5$), spectral clustering performed perfectly in 28/30 14-node networks (**A,B**) and in 29/30 16-node networks (**C,D**). For the random networks ($p = 1$), spectral clustering performed perfectly in all 14-node networks (**A,B**) and in 29/30 16-node networks (**C,D**). Red squares are the mean across tested networks, and blue bars indicate standard error of the mean. Overall, these results show that spectral clustering can accurately identify the MIB in a variety of network types.
