# Supplementary material for: Information integration in large brain networks
Source: PLoS Comput Biol. 2019 Feb 7;15(2):e1006807. doi: 10.1371/journal.pcbi.1006807 (PMC6382174; doi:10.1371/journal.pcbi.1006807)
Supplement: S7 Fig — (PDF) [file pcbi.1006807.s008.pdf]

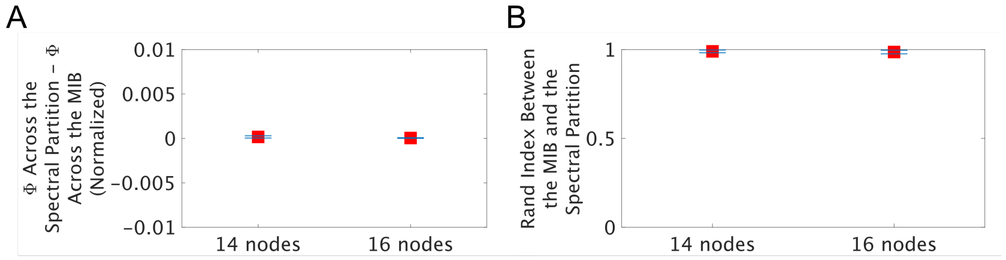

S7 Figure. Spectral clustering accuracy in small autogregressive systems. In the main body of the paper, we showed that our spectral clustering-based approach finds the exact MIB (determined through a brute-force search) in almost all small brain-like networks of coupled oscillators (Fig. 2). To show that our approach not only generalizes across a variety of network types (Fig. S6), but also generalizes across a variety of network *dynamics*, we also used the same networks used in Fig. 2 to generate a set of autoregressive time-series data (as opposed to coupled Rössler oscillators). Autoregressive data are, by construction, multivariate Gaussian, and so the same estimator of geometric integrated information used for the Rössler oscillators can be used for autoregressive simulations. To ensure that the autoregressive simulations were linearly stable, we used `var_specrad.m` function of the MVGC Multivariate Granger Causality Matlab Toolbox **?** to decay the coefficients of the adjacency matrices of the brain-like networks of Fig. 2, so that their spectral radii were 0.8. Moreover, because the model order of our simulations was 1, we used a time-lag of 1 in our calculations of integrated information for these data. Our spectral clustering approach once again performed almost perfectly: the difference to the ground-truth values of Φ^{G} was 0 for 48/50 of both the 14- and 16-node networks (**A**), and the Rand Index between the spectral partitions and the ground-truth MIBs was 1 for the same 48/50 14- and 16-node networks (**B**). Just as was the case for the coupled oscillators, the Queyranne algorithm performed poorly for the autoregressive data, yielding no exact matches for the 14-node networks (mean difference to the ground-truth normalized Φ^{G} values = 0.04 bits, mean Rand index = 0.56) and only one exact match in the 16-node networks (mean difference to the ground-truth normalized Φ^{G} values = 0.03 bits, mean Rand index = 0.57).
