# Supplementary material for: Information integration in large brain networks
Source: PLoS Comput Biol. 2019 Feb 7;15(2):e1006807. doi: 10.1371/journal.pcbi.1006807 (PMC6382174; doi:10.1371/journal.pcbi.1006807)
Supplement: S8 Fig — (PDF) [file pcbi.1006807.s009.pdf]

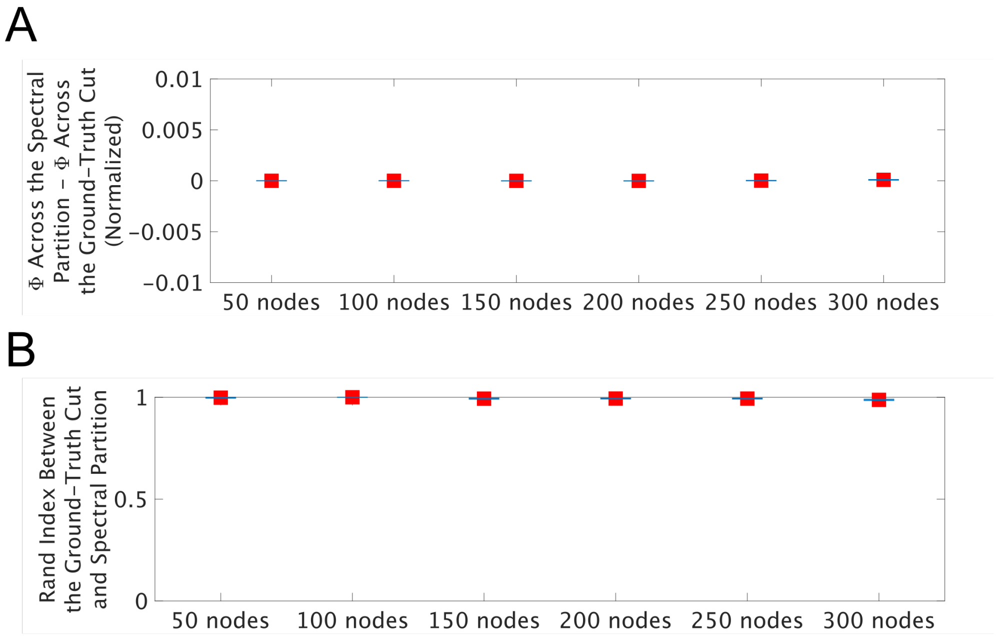

S8 Figure. Spectral clustering accuracy in large autogregressive systems. In the main body of the paper, we showed that our spectral clustering-based approach well approximates the MIB of large-cut networks (Fig. 3). To further show that our approach generalizes across a variety of network dynamics, we also used the same large, cut networks used in Fig. 3 to generate a set of autoregressive time-series data, following the same procedure as in Fig. S7. We found that our spectral clustering approach performed almost perfectly in large autoregressive systems: the difference between Φ^{G} across the spectral partitions and Φ^{G} across the ground-truth cuts was 0 for 191/240 networks tested, with the difference less than 10^{-6} bits (normalized) for all network sizes. The spectral partitions were perfect matches to the ground-truth cuts for those same 191/240 networks, with a mean Rand index greater than 0.98 for all network sizes.
