# Supplementary material for: Information integration in large brain networks
Source: PLoS Comput Biol. 2019 Feb 7;15(2):e1006807. doi: 10.1371/journal.pcbi.1006807 (PMC6382174; doi:10.1371/journal.pcbi.1006807)
Supplement: S9 Fig — (PDF) [file pcbi.1006807.s010.pdf]

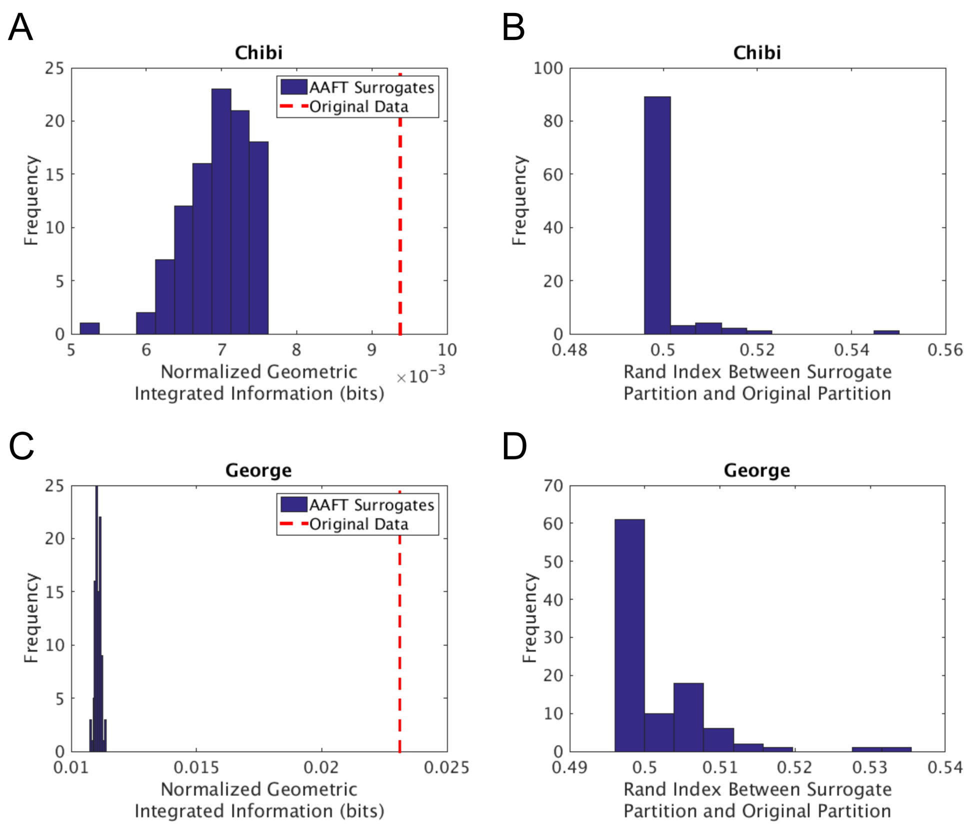

S9 Figure. Surrogate analysis of monkey ECoG results. To test the robustness of our results for the full monkey brains (Fig. 4), we used surrogate statistical testing. We generated 100 surrogates for each ECoG electrode in both monkeys using the Amplitude Adjusted Fourier Transform (AAFT) algorithm **?**, which creates a time-series with the same linear structure and amplitude distribution as the original data, but which is otherwise random. We created surrogates from each electrode independently so as to break any cross-electrode coupling or correlations. We thus had 100 surrogate multivariate datasets for each monkey. We then calculated integrated information in each of these datasets using our spectral clustering approach. If the results reported in Fig. 4 are meaningful, i.e. they are not an artifact of either the ECoG data or the spectral clustering algorithm, then we should expect two results here: 1) normalized integrated information in the original ECoG data (in which there is some information actually being integrated) should be significantly greater than the distribution of integrated information calculated from the surrogate datasets (in which there is objectively no information integration, despite sharing all linear features with the original data), and 2) the estimated MIBs of the surrogate datasets should be random, and thus dissimilar to the estimated MIBs of the monkey cortices, which we claim are not random (Fig 4). That is precisely what we found: normalized integrated information was significantly higher in the original data than in the surrogate data in both monkeys (**A,C**), and the Rand indices between the MIBs estimated from original data and the MIBs estimated from the surrogate data clustered around 0.5 in both monkeys (**B,D**), which is precisely what we would expect for random partitions.
